# Supplementary material for: Bulk flow of cerebrospinal fluid observed in periarterial spaces is not an artifact of injection
Source: eLife. 2021 Mar 9;10:e65958. doi: 10.7554/eLife.65958 (PMC7979157; doi:10.7554/eLife.65958)
Supplement: Figure 3—source data 1. [file elife-65958-fig3-data1.docx]

Data for Figure 4:

| Figure 4a: Dual-syringe | Figure 4a: Single-Injection |
| --- | --- |
| t_DS,vrms_DS,sem_DS | t_SI,vrms_SI,sem_SI |
| 1.6667e-06,24.396,4.2948  0.16667,24.485,1.8614  0.33333,18.809,6.6238  0.5,21.275,4.937  0.66667,23.093,5.6759  0.83334,23.936,5.3225  1,25.392,6.5749  1.1667,24.969,11.092  1.3333,18.562,7.0391  1.5,23.099,8.253  1.6667,23.309,5.7293  1.8333,17.676,6.7626  2,21.238,2.8873  2.1667,23.14,3.8919  2.3333,21.733,5.9457  2.5,18.493,2.1268  2.6667,20.386,4.9332  2.8333,19.683,3.8837  3,16.783,2.7501  3.1667,18.335,6.7701  3.3333,17.872,7.2307  3.5,18.501,4.4492  3.6667,18.103,5.4238  3.8333,19.92,6.9978  4,19.034,5.969  4.1667,19.291,8.1194  4.3333,17.496,7.3432  4.5,21.59,3.4973  4.6667,22.86,7.697  4.8333,23.552,7.4845  5,21.578,5.241  5.1667,23.596,6.1527  5.3333,21.892,2.7799  5.5,21.415,2.3636  5.6667,21.153,4.4934  5.8333,20.539,3.4338  6,19.749,5.0256  6.1667,19.649,1.4319  6.3333,15.978,3.2491  6.5,20.205,4.7592  6.6667,17.948,4.0333  6.8333,19.585,4.5981  7,19.898,7.2985  7.1667,17.378,4.8777  7.3333,17.939,5.9758  7.5,16.712,4.4949  7.6667,19.447,7.2457  7.8333,19.639,4.9181  8,20.58,5.859  8.1667,19.416,6.5866  8.3333,15.604,4.9133  8.5,17.132,4.7161  8.6667,17.545,4.2283  8.8333,20.215,6.1433  9,19.048,6.4378  9.1667,18.731,3.7849  9.3333,18.33,4.1351  9.5,16.641,4.1143  9.6667,18.169,6.1708  9.8333,18.519,4.3253  10,19.205,3.156  10.167,17.192,4.3326  10.333,19.687,4.6381  10.5,17.973,5.0972  10.667,20.195,4.8126  10.833,17.6,3.1967  11,20.012,6.4241  11.167,18.318,5.4346  11.333,20.684,8.2049  11.5,22.232,6.1378  11.667,21.616,5.2168  11.833,21.103,4.595  12,24.626,4.4966  12.167,23.19,6.7231  12.333,20.619,3.242  12.5,19.271,6.25  12.667,20.209,2.9255  12.833,20.989,5.8293  13,19.593,7.139  13.167,20.093,6.8745  13.333,16.345,5.4292  13.5,15.247,4.8003  13.667,16.522,5.23  13.833,17.782,5.9532  14,15.228,5.5376  14.167,19.545,6.1894  14.333,20.493,7.2843  14.5,22.797,4.4951  14.667,17.32,6.6372  14.833,17.709,5.0643  15,19.934,7.4689 | 1.6667e-06,26.672,12.385  0.16667,24.678,9.3133  0.33333,26.221,7.0994  0.5,27.442,7.6166  0.66667,26.182,5.0134  0.83334,28.759,5.6983  1,24.238,3.5492  1.1667,25.767,5.2583  1.3333,27.814,5.7499  1.5,28.131,6.3098  1.6667,25.521,5.7819  1.8333,23.409,4.5066  2,24.372,4.9166  2.1667,22.507,7.3014  2.3333,23.567,4.6686  2.5,24.399,5.7327  2.6667,26.139,5.676  2.8333,21.633,3.7195  3,24.453,3.7181  3.1667,25.245,5.8917  3.3333,24.593,5.3078  3.5,25.07,5.9608  3.6667,25.139,4.7809  3.8333,27.647,7.2751  4,25.101,4.6499  4.1667,23.791,6.184  4.3333,20.115,3.776  4.5,24.306,4.1251  4.6667,22.334,4.6664  4.8333,21.499,6.3892  5,20.02,4.119  5.1667,21.179,3.092  5.3333,23.014,5.4334  5.5,22.066,2.7387  5.6667,20.959,3.1511  5.8333,20.981,4.829  6,23.02,4.8268  6.1667,20.233,4.1309  6.3333,18.898,3.4695  6.5,18.883,1.7117  6.6667,17.912,3.4703  6.8333,18.915,5.0381  7,21.076,1.4898  7.1667,18.893,3.8821  7.3333,22.128,7.58  7.5,20.825,4.8033  7.6667,20.793,3.7812  7.8333,20.378,8.7467  8,23.125,0.96148  8.1667,21.168,4.8129  8.3333,19.8,2.7952  8.5,19.13,1.8897  8.6667,18.452,4.7198  8.8333,20.026,4.0091  9,21.27,2.5469  9.1667,21.084,1.9925  9.3333,22.508,1.7414  9.5,20.526,2.6773  9.6667,19.574,5.4054  9.8333,20.803,5.2803  10,23.165,2.1386  10.167,19.753,2.2725  10.333,17.144,5.8147  10.5,18.235,5.4426  10.667,18.711,3.987  10.833,23.65,0.63703  11,19.421,5.9873  11.167,20.004,4.33  11.333,20.294,6.0188  11.5,18.742,4.84  11.667,20.159,7.6519  11.833,21.798,7.4636  12,19.352,3.4684  12.167,21.148,3.8698  12.333,17.397,5.2935  12.5,18.541,5.0172  12.667,19.698,4.5488  12.833,21.042,3.4543  13,19.705,1.6537  13.167,17.753,4.2601  13.333,20.774,2.8593  13.5,18.946,3.1307  13.667,19.894,4.2858  13.833,19.43,5.1934  14,18.369,3.9047  14.167,19.037,3.275  14.333,21.086,1.6678  14.5,21.689,2.2238  14.667,23.388,2.2227  14.833,24.011,0.093615  15,21.047,6.4477 |

Figure 4d: Mean flow speed (mfs) comparison

SI - single injection

DS - dual syringe

mfs_SI,mfs_DS

22.667,14.09

7.81,20.94

13.83,23.94

13.74,20.94

20.76,6.5

15.71,19.63

Figure 4e: Backflow fraction (bff) comparison

SI - single injection

DS - dual syringe

bff_SI,bff_DS

0.061234,0.27714

0.15539,0.085151

0.20291,0.37079

0.36142,0.09499

0.41266,0.41372

0.39185,0.2075
