# Supplementary material for: Bulk flow of cerebrospinal fluid observed in periarterial spaces is not an artifact of injection
Source: eLife. 2021 Mar 9;10:e65958. doi: 10.7554/eLife.65958 (PMC7979157; doi:10.7554/eLife.65958)
Supplement: Figure 4—source data 1. [file elife-65958-fig4-data1.docx]

Source data for Figure 5i (Comparing delay times, delta T)

Figure 5i:SI - single injection, DS - dual syringe

delt_SI,delt_DS

0.041,0.049

0.047,0.063

0.053,0.054

0.039,0.061

0.057,0.043

0.043,0.042
